# Chronic Kidney Disease in Balkan Countries—A Call to Action for Timely Diagnosis and Monitoring

**DOI:** 10.3390/diagnostics12092162

**Published:** 2022-09-06

**Authors:** Igor Mitić, Mario Laganović, Ivelina Marinova, Nina Gancheva, Valentina Nakić, Dragana Melentijevic, Emil Paskalev, Rajko Vajd, Andrej Škoberne

**Affiliations:** 1 Medical Faculty, University of Novi Sad, Hajduk Veljkova 3, 21137 Novi Sad, Serbia; 2 Clinic of Nephrology and Clinical Immunology, Clinical Centre of Vojvodina, Hajduk Veljkova 1, 21137 Novi Sad, Serbia; 3 Department of Internal Medicine, University Hospital Merkur, Zajčeva 19, 10000 Zagreb, Croatia; 4 Clinic of Nephrology and Transplantation, University Hospital “Alexandrovska”, Sveti Georgi Sofiyski 1, 1431 Sofia, Bulgaria; 5 Medical Diagnostic Center 1, Ploshtad Otets Paisiy 40, 9000 Varna, Bulgaria; 6 Health Center Gajnice, Mahatme Gandhija 5, 10090 Zagreb, Croatia; 7 Public Health Center “Dr Simo Milosevic”, Pozeska 82, 11000 Belgrade, Serbia; 8 Community Health Centre Medvode, Ostrovrharjeva Ulica 6, 1252 Medvode, Slovenia; 9 Department of Nephrology, University Medical Centre Ljubljana, Zaloška cesta 7, 1000 Ljubljana, Slovenia; 10 Medical Faculty, University of Ljubljana, Vrazov Trg 2, 1000 Ljubljana, Slovenia

**Keywords:** chronic kidney disease, CKD, screening, awareness, diagnosis, monitoring, call to action, Balkan, Eastern Europe

## Abstract

Chronic kidney disease (CKD) is a serious illness with important consequences for patients and health systems. Estimation of prevalence and incidence, especially in early stages, is difficult due to a lack of epidemiological studies and consolidated registries. In general, the disease awareness is low, and thus CKD is not timely diagnosed in most cases. Robust screening programs are not implemented in Eastern European countries. A panel consisting of Primary Care Physicians and Nephrologists from Bulgaria, Croatia, Serbia, and Slovenia virtually met in December 2021 to discuss current CKD awareness and diagnostic approaches in the Balkan area The meeting resulted in specific calls to action in the region to improve the number and quality of epidemiology studies and the level of awareness among patients and medical communities, as well as implementation of screening programs in high-risk populations. Collaboration between specialists was acknowledged as a crucial driver for optimal management of patients with CKD. Joint efforts are required to persuade healthcare authorities to establish specific policies for better care of kidney patients.

## 1. Introduction

Chronic kidney disease (CKD) is a severe condition affecting approximately 800 million people (10%) of the world’s population [1], out of which 75–100 million are in Europe [2,3]. An accurate estimation of its prevalence and incidence, especially in the initial stages, is complicated by the lack of epidemiologic studies in the region. Decline of kidney function may be asymptomatic in early stages and probably many years after, thus leaving it far from the spotlight of public and medical communities’ attention. CKD is currently considered one of the most neglected chronic diseases [4].

Due to the increasing incidence of cardiovascular (CV) disease and diabetes in an aging population, representing the high-risk populations, the progression to end-stage renal disease (ESRD) is estimated to increase by 5–8% each year [5]. However, the advanced stages requiring dialysis or other renal replacement therapies are only a part of the problem. Of 100 persons with CKD, only 1 will require dialysis or transplantation [3,4]. Due to its enormous effect on heightening the risk of CV diseases and events, CKD ranked as the 10th leading cause of death globally in 2019 [1]. If appropriate measures are not taken urgently, the projection is that CKD will rise among the top 5 causes of death by 2040, ahead of all cancer types and diabetes [4]. Mortality rates are rising from the earlier stages (estimated glomerular filtration rate [eGFR] <60 mL/min/1.73m^2^ or normal eGFR with albuminuria) [6,7] and add to the mortality risk of the associated diseases.

Disease awareness among patients is low. A recent paper from Germany showed that almost 80% of patients diagnosed with CKD at an early stage and 30% in the later stages were not aware of their disease [8]. A study performed in Poland [9] in an elderly population showed a prevalence of CKD of 29.4% with a level of disease awareness of only 3.2%. In the United States, 9 out of 10 people with CKD do not know they have this disease [10]. Gaps in education about the diagnosis and the implications may be due to inefficient testing, communication of the results or understanding of the information.

Although screening of the general population is debatable [11,12], the regular assessment of serum creatinine and albuminuria in high-risk groups (elderly patients with arterial hypertension, diabetes, cardiovascular diseases or familial history of CKD) was suggested as a more cost-efficient approach. Most Western European countries have implemented national screening programs for CKD in patients with diabetes and hypertension [1], but screening remains suboptimal in some countries in Central and Eastern Europe (CEE) due to limited infrastructure and resources, social, educational, and cultural differences [13].

The alarming rise in prevalence and mortality rate linked to CKD in the CEE region [2,13] requires an immediate call to action aiming to increase disease prevention and its early identification and treatment. The gaps that should be addressed urgently include development of structured simple screening, a diagnostic and treatment algorithm on a national and/or regional level endorsed by professional societies and designed for implementation in routine practice. In this manuscript we present the Balkan initiative focused on screening and diagnosis of CKD in the region.

## 2. Materials and Methods

Driven by the need to challenge the status quo, a panel consisting of eight experts involved in the management of patients with CKD from four countries in CEE virtually met in December 2021 to discuss the need to improve CKD awareness and diagnosis in the Balkan area. Four primary care professionals (PCPs) and four nephrologists from Bulgaria, Croatia, Serbia, and Slovenia participated in this meeting. We divided the CKD issues into three sections that were discussed independently at the meeting: epidemiology, risk factors and disease awareness, diagnosis, and treatment. For each of these topics, representatives described the current situation in their respective countries and fields of profession. This included relevant data from each country, the current state of the healthcare system in general, and the fields of primary care and nephrology in particular, including relevant logistical and financial issues. Relevant legal and insurance issues were also discussed. After these initial presentations, overlapping issues that were common in all of the participating countries were identified and written down in order to summarize the key points of the current status of each topic. Afterwards, a framework for tackling these challenges and problems was discussed and key messages were formed in order to form the specific calls to action for each topic. All of the points presented in the results were included after unanimous agreement among the participants. Our main aim was to identify and prioritize key challenges in the management of CKD and to provide possible solutions that would be relevant in our region and possibly beyond in similar countries.

## 3. Results

### 3.1. Epidemiology

#### 3.1.1. Current Status

Robust epidemiology data are lacking in the region. We extracted CKD prevalence rates in the Balkan countries from a recent international publication [3] that had established these figures using different data sources (published literature, vital registration systems, end-stage kidney disease registries, and household surveys). In the past 30 years, the change in CKD prevalence rates in the participating Balkan countries ranged from −1.1% to 2.6%, whereas the death rates due to CKD increased up to 45%, except for Slovenia, where the calculated reduction of mortality rate was approximately 30% (Table 1).

This is most likely related to the reduction of CV mortality in Slovenia, where the age-standardized CV mortality rate had dropped from 557.4 deaths per 100,000 population in 1990 to 222.4 deaths per 100,000 population in 2017 in men, and from 371.5 deaths per 100,000 population in 1990 to 153.1 deaths per 100,000 population in 2017 in women [14,15]. A similar improvement in CV mortality has been observed when the whole population of the EU region is considered.

Nevertheless, incidence, prevalence, and mortality rates by CKD stage and other relevant factors (age, comorbidities) are not available in the region. Published literature indicates a higher prevalence of CKD stages G3–G5 and a lower prevalence of renal replacement therapies in Eastern Europe compared to Western countries [16]. The difference cannot be fully explained by the difference in mortality rates or the incidence of different risk factors for reduced kidney function.

#### 3.1.2. Call to Action

- Design and implement an epidemiology study across the Balkan region to collect standardized data on the incidence and prevalence of early stages and causes of CKD. A unique research protocol and uniform data collection and reporting would allow building real-world evidence across the Balkan region, further exploring the variability across countries and informing management strategies based on reliable data.
- Expand existing reporting protocols and registries to include all CKD patients, irrespective of stage of the disease.
- Discuss possibilities to extract retrospective data (serum creatinine and albuminuria) from electronic files and use them as benchmark for epidemiology studies.

### 3.2. Risk Factors and Disease Awareness

#### 3.2.1. Current Status

In general, the level of CKD awareness is low in the general population and variable in the medical community. Usually, educational information related to kidney diseases are provided annually in different media related to World Kidney Day, by representatives of national professional medical societies. In 2021, the campaigns and webinars for patients organized by the Croatian Society of Nephrology were appreciated by the international officials and included in the Champions’ Report [17]. In Slovenia, high awareness in the medical community is maintained through regular activities dedicated to primary care physicians and medical residents. Despite increasing of online communications for different health conditions during the Coronavirus disease-19 (COVID-19) pandemic, CKD did not gain a higher visibility in the region.

#### 3.2.2. Call to Action

- Advocate for development and implementation of professional awareness campaigns for patients and medical professional communities.
- Increase media presence of national key opinion leaders to enhance education regarding primary and secondary prevention of CKD.
- Address specifically high-risk populations and collaborate with patient organizations and medical societies to improve recognition of chronic conditions that are risk factors for CKD.
- Promote a unitary and coherent communication strategy to European and national professional societies to streamline education of their members during regular scientific meetings and webinars.

### 3.3. Diagnosis and Treatment

#### 3.3.1. Current Status

Diagnosis of CKD is based on a persistent reduction of eGFR and/or markers of kidney damage, including high urine albumin-creatinine ratio (UACR) or urine protein-creatinine ratio (UPCR) (Table 2) [18]. 

No general CKD screening programs aimed at identifying the presence of the disease in the general population without risk factors are currently available in any of the four participating countries. However, serum creatinine and albuminuria or proteinuria are routinely measured in high-risk patients, like those with diabetes, arterial hypertension or CV diseases. For chronic patients, annual blood and urine analysis are reimbursed in all countries, however no information could be provided on the actual assessment in overall or high-risk populations. Nevertheless, in the past years, the amount of laboratory testing decreased due to reluctance to physically access the healthcare facilities induced by COVID-19. Despite being long recognized by healthcare professionals as an unmet need, the screening protocols for CKD are currently not being employed successfully.

In all countries in the Balkan region, CKD is diagnosed by PCP or other specialist (e.g., endocrinologist, cardiologist, urologist, or internal medicine specialist) and confirmed, when needed, by the nephrologist. Most patients are referred to a nephrologist in the later stages, mainly for complications and assessment of eligibility for renal replacement therapies. Major challenges in CKD management and gaps in the referral pathway are summarized in Table 3. Despite differences in monitoring strategies adopted in each country, medical and laboratory follow-up is usually performed twice per year, the frequency depending on the severity of the CKD and comorbidities.

Current pharmacologic approaches in CKD include angiotensin-converting enzyme (ACE) inhibitors, angiotensin receptor blockers (ARBs), and diuretics. The level of reimbursement is variable (from 25–50% in Bulgaria to 100% in Croatia and Slovenia). In 2021, dapagliflozin was granted approval in Europe for the treatment of adults with CKD [19] irrespective of diabetes status, becoming the first sodium-glucose transporter 2 (SGLT2) inhibitor with this indication. Slovenia is the only country in the region where dapagliflozin is reimbursed for the indication in CKD. In general, the treatment is initiated by nephrologists according to local and international guidelines and continued by PCPs based on the national protocols however, initiation by PCPs is possible and should be encouraged in situations where reimbursement is provided in this setting.

#### 3.3.2. Call to Action

**Figure 1:**
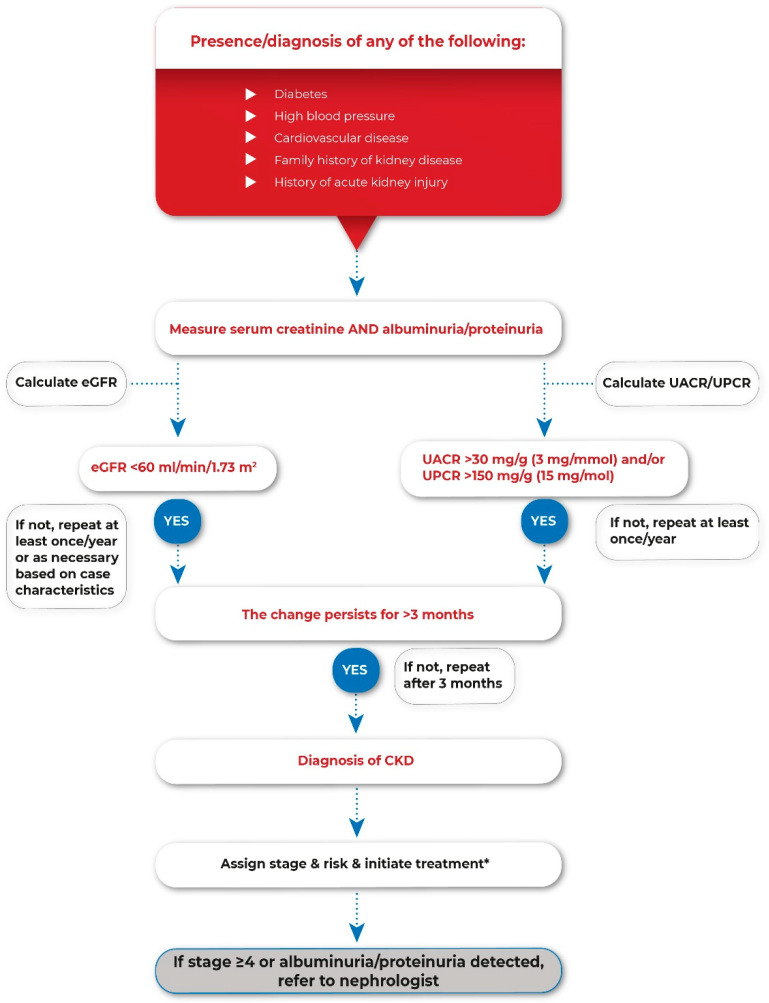
Screening algorithm of CKD in high-risk population. CKD, chronic kidney disease; eGFR, estimated glomerular filtration rate; UACR, urinary albumin-creatinine ratio; UPCR, urinary protein-creatinine ratio. * according to locally available protocols and guidelines.

- Develop and implement screening programs in high-risk populations to allow diagnosis in early stages and adequate management to slow the decline of renal function (Figure 1).
- Develop simple algorithms for risk assessment, diagnosis, and referral to nephrologists.
- Include CKD risk assessment and diagnostic criteria in all relevant communications, and scientific meetings and webinars.
- Promote reporting on laboratory results of eGFR and UACR/UPCR in all patients with serum creatinine and albuminuria or proteinuria assessment.
- Create multidisciplinary working groups (nephrologist, endocrinologist, cardiologist, PCP) to coordinate implementation of CKD-related activities and lead high-level discussions to include CKD on the agenda of decision makers.
  ○ Create the framework for structured collaboration of multidisciplinary team in national and regional kidney networks
  ○ Empower PCPs to initiate and monitor treatment in CKD
  ○ Increase number of nephrologists to treat CKD in all stages
  ○ Provide reimbursement of treatments with strong and reliable results on mortality and renal function
  ○ Share best practices of diagnosis and treatment between countries and institutions
  ○ Collaborate with specialists in pharmaco-economics to assess the cost-effectiveness of early diagnosis and timely treatment in CKD

## 4. Discussion

CKD is an emerging condition that will have a major impact on public health in most countries around the world. In parallel with the rise of incidence of CKD, we are also witnessing significant progress in diagnostic methods and therapeutic approaches. New biomarkers have been discovered that could enable a more reliable and accurate identification of kidney disease patients, particularly those with a high risk of progression [20,21]. New technologies are also being employed to tackle the problem of CKD. Nanotechnology is being used in research settings as a tool to identify and treat diverse kidney diseases [22,23] along with new genetic technology [24,25]. New treatment strategies are also emerging. The recognition of the importance of complement activation for progression of many kidney diseases has led to the development of a number of new medications targeting complement pathways [26]. The recognition that mitochondria play an important role in kidney diseases has also led to new paradigms for CKD treatment [27,28]. In spite of all this exciting progress, we are still insufficient in the most basic aspects of CKD management—early diagnosis and early treatment with therapy that is already available.

This is the first meeting where experts from Balkan countries discussed the challenges in CKD awareness, diagnosis and treatment, and outlined key points of specific calls to action. CKD remains undiagnosed to a large extent in Balkan countries. Although the general understanding is that severity comes with higher stages, the public and most medical communities are not informed about negative consequences and mortality risk in early stages. Despite economic and cultural differences between countries, main unmet needs in the region are similar and related to education, awareness, epidemiology studies, screening protocols, and simple algorithms for diagnosis and referral.

The onset and progression of CKD can be prevented. Efficient elimination and control of risk factors should be one main goal of education and clinical interventions [29] to delay worsening of the disease and mortality. The associated costs in late stages of the disease are considered “catastrophic” for the healthcare systems, therefore early detection and management are crucial.

Each action suggested for improvement of CKD management requires consistent efforts and a strategic approach. The experts will act as leading voices in each country and in the region to gather resources and knowledge to plan, develop, and implement available guidelines and best practices adapted to the local context. Additionally, multidisciplinary teams should be involved in creation and enhancement of kidney networks both locally and regionally to delay and prevent disease progression. Kidney health should be brought in the focus of public health authorities, medical professional societies, and general population to improve screening, diagnosis, and timely treatment of this chronic disease with significant consequences. Finally, we believe that cooperation amongst different countries in order to share experience on practical approaches that actually work in real-life situations is one of the best ways of tackling the reality of an emerging CKD epidemic.

## Figures and Tables

**Table 1:** CKD prevalence and CKD-related mortality rates in the four countries of the Balkan region, as of 2017.

*Country/Region*			
Bulgaria	***Count***	***Age-Standardized*** ***Rate per 100,000***	***Percentage Change in Age-Standardized Rates between 1990 and 2017***
	**Prevalence (95% UI)**		
	981,339 (909,610 to 1,061,687)	8000 (7420 to 8630)	2.6% (−0.7 to 5.9)
Croatia	562,778 (520,865 to 610,153)	7779 (7206 to 8390)	1.2% (−3.1 to 6.2)
Serbia	1,142,513 (1,063,208 to 1,237,929)	8421 (7846 to 9069)	−0.5% (−4.1 to 3.3)
Slovenia	266,527 (247,205 to 289,578)	7581 (7056 to 8179)	−1.1% (−5.7 to 3.2)
**Central Europe**	**13,951,402** **(12,930,450 to 15,136,020)**	**7659** **(7115 to 8282)**	**−2.7%** **(−6.2 to 1.4)**
	**Mortality (95% UI)**		
Bulgaria	1447 (1346 to 1557)	10.1 (9.4 to 10.8)	45.7% (35.3 to 57.2)
Croatia	829 (776 to 888)	8.8 (8.2 to 9.4)	35.3% (25.6 to 46.0)
Serbia	2386 (1982 to 2607)	14.8 (12.4 to 16.1)	18.8% (7.3 to 32.0)
Slovenia	213 (195 to 232)	4.4 (4.0 to 4.8)	−30.2% (−36.7 to −23.1)
**Central Europe**	**16,284** **(15,806 to 16,706)**	**7.5** **(7.3 to 7.7)**	**−21.2%** **(−23.6 to −18.9)**

UI, uncertainty interval. Thes data had been taken from [3].

**Table 2:** Criteria for diagnosis (**A**) and staging (**B**) of CKD.

**A. Diagnosis of CKD**	
**One or more markers of kidney damage present for >3 months**	Albuminuria (UACR >30 mg/g [>3 mg/mmol]) or proteinuria (UPCR > 150 mg/g [> 15 mg/mmol]) Urine sediment abnormalities Electrolyte and other abnormalities due to tubular disorders Structural abnormalities detected by imaging and/or biopsy
**OR**	
**eGFR <60 mL/min/1.73 m^2^ persistently present >3 months**	
**B. Staging of CKD**	
**Stage**	**eGFR values (mL/min/1.73 m^2^)**
G1	≥90
G2	60–89
G3a	45–59
G3b	30–44
G4	15–29
G5	<15

CKD, chronic kidney disease; eGFR, estimated glomerular filtration rate; KDIGO, Kidney Disease: Improving Global Outcomes; UACR, urinary albumin-creatinine ratio; UPCR, urinary protein-creatinine ratio.

**Table 3:** Current challenges in diagnosis and treatment of CKD in the Balkan countries.

Challenges in Diagnosis
Limited access to healthcare systems during COVID-19 pandemic Limited reimbursement of serum creatinine and albuminuria/proteinuria testing in some cases Quantitative testing of albuminuria and proteinuria is not available in some settings of primary care No uniform frequency assessment of serum creatinine and albuminuria/proteinuria in high-risk population
**Challenges in Treatment**
Lack of reimbursement of SGLT2 inhibitor (dapagliflozin) in CKD without diabetes in some cases Low number of nephrologists available for referral and CKD treatment
**Gaps in the Referral Pathway**
A limit on the number of referrals by PCP per year to nephrologist in some cases Limited timely referral from endocrinologists, cardiologists, and other specialties treating high-risk patients Long waiting times from referral to secondary/tertiary care No clear and simple algorithms of referral indication based on cut-of eGFR and UACR/UPCR Kidney health network is not available or insufficiently developed

CKD, chronic kidney disease; eGFR, estimated glomerular filtration rate; SGLT2—sodium-glucose transporter 2; UACR, urinary albumin-creatinine ratio; UPCR, urinary protein-creatinine ratio.

## Data Availability

Table 1 includes data taken without any change from a published source (GBD Chronic Kidney Disease Collaboration. Global, regional, and national burden of chronic kidney disease, 1990–2017: a systematic analysis for the Global Burden of Disease Study 2017. *Lancet.* **2020**, *395*, 709–733. doi: 10.1016/S0140-6736(20)30045-3). Permission from Elsevier to use the data has been obtained prior to manuscript submission (Creative Commons Attribution License available at: https://creativecommons.org/licenses/by/4.0/, last accessed on 16 June 2022).

## Abbreviations

CEE—Central and Eastern Europe, CKD—chronic kidney disease, CV—cardiovascular, eGFR—estimated glomerular filtration rate, ESRD—end-stage renal disease, EU—European Union, KDIGO—Kidney Disease: Improving Global Outcomes, PCP—primary care professional, SGLT2—sodium-glucose transporter 2, UACR—urinary albumin-creatinine ratio, UI—uncertainty interval, UPCR—urinary protein-creatinine ratio.
